# A case report of a dramatic response to olaparib in a patient with metastatic pancreatic cancer harboring a germline BRCA2 mutation

**DOI:** 10.1097/MD.0000000000017443

**Published:** 2019-10-04

**Authors:** Huan Wang, Chenyu Mao, Ning Li, Liping Sun, Yulong Zheng, Nong Xu

**Affiliations:** a Department of Medical Oncology, First Affiliated Hospital of Zhejiang University, Shangcheng District, Hangzhou; b Department of Pathology, Shaoxing Hospital of Zhejiang University/Shaoxing People's Hospital, Shaoxing, People's Republic of China.

**Keywords:** BRCA mutation, clinical trial, olaparib, target therapy

## Abstract

**Rationale:**

Pancreatic cancer (PC) is considered as one of the deadliest cancers all over the world. Germline and somatic BRCA1/2 mutations have been widely studied in breast and ovarian carcinomas as they have been found to enhance the risk for disease progression. Olaparib, an oral poly(adenosine diphosphate-ribose)polymerase (PARP) inhibitor, has been approved for the treatment strategy of ovarian cancer with any BRCA1/2 mutations. There is a lack of studies which focus on the treatment of other cancer with BRCA-Mutation.

**Patient concerns:**

This report describes a patient whose presenting complaints were “Physical examination showed that the pancreas was occupied for one month.” He initially was diagnosed with stage IV PC based on conventional imaging and pathologic assessment. He had a known germline BRCA 2 mutation, which exhibited a good response to PARP inhibitor therapy.

**Diagnosis:**

Through the biopsy histopathological examination, imaging examination, and genetic testing, the patient was diagnosed as metastatic PC with BRCA2 mutation.

**Interventions:**

He received gemcitabine and albumin-bound paclitaxel chemotherapy from March 15, 2017 to June 30, 2017, and Nivolumab immunotherapy as the maintenance therapy. After serum CA-199 level increased, Olaparib was orally administered from August 17, 2017 to March. After tumor relapsed, he received multiple lines of chemotherapy, including Trametinib Oxaliplatin, S-1, bevacizumab, and irinotecan liposome injection till July 17, 2018.

**Outcomes:**

We observed the patient had a good progression-free survival (7.4 months); the lesion of the pancreas was classified as partial disease through Olaparib treatment, which indicated significant shrinkage. But it is difficult to conclude whether such therapy could help prolong the overall survival for such patients.

**Lessons:**

The targeted therapy Olaparib showed early signs of potential in treating PC in patients with mutations of the BRCA genes. With emerging therapeutic modalities and next-generation sequencing development, it is increasingly relevant to consider mutation screenings of patients with PC.

## 1 Introduction

Pancreatic cancer (PC) is a highly lethal disease in malignant tumors. The mortality of PC is quite similar to its incidence. There are often no symptoms until the disease reaches an advanced stage for most patients with PC. The scientific data of 5-year survival rate for PC is <5% when all stages are combined.^[1]^ PC can be treated with surgery, radiotherapy, chemotherapy, palliative care, or a combination of these.^[2]^ The current National Comprehensive Cancer Network recommendations suggest acceptable chemotherapy combinations for the patient, which included FOLFIRINOX, gemcitabine combining nab-paclitaxel, and gemcitabine combining erlotinib.^[3]^ The development of molecular targeted therapy is also supposed to be a potential treatment direction for the treatment of PC.

BRCA1/2, a cancer highly susceptibility gene, has been found in breast and ovarian cancer as mutation carriers widely. Studies of unselected patients with PC have detected BRCA1/2 mutations at a frequency of 4% to 7%.^[4,5]^ For PC, the estimated cumulative risk by the age of 80 years for male subjects was 6.9% (95% confidence interval [CI] 3.8–10.0) and for female subjects 2.8% (0.9–4.7).^[6]^ BRCA1 and BRCA2 proteins are significant for high-fidelity repair of double-strand breaks of DNA by the homologous recombination repair (HRR) pathway. Deficiency in HRR is a key target for PARP inhibitors.^[7]^ Olaparib has been approved as an orphan drug designation by the FDA for the treatment of patients with PC.

## 2 Case report

**Figure 1:**
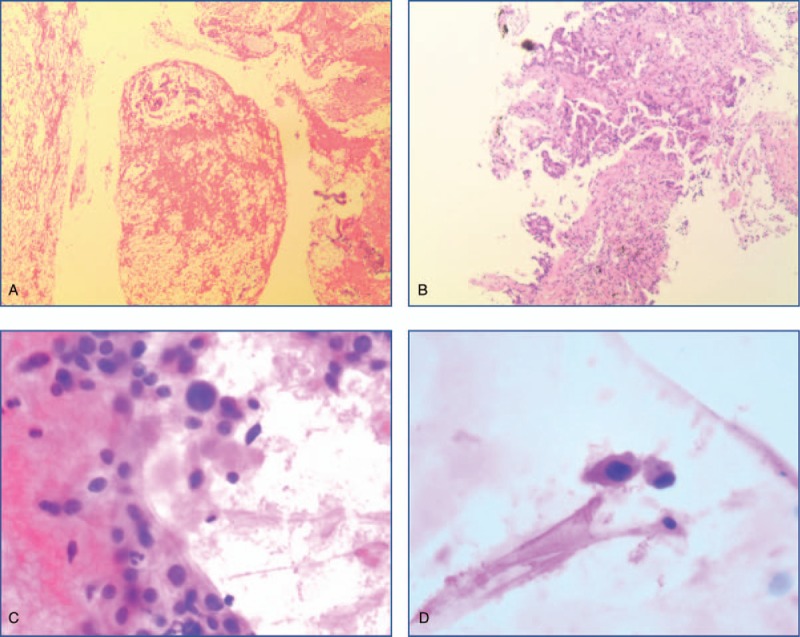
(The wax block of pancreatic specimen, original magnification ×100): HE stain of irregular tubular or adenoid structures in the heteromorphic cells (A); (the wax block of lung specimen, original magnification ×100): HE stain of irregular tubular or adenoid structures formed by heteromorphic cells in the fibrous interstitial structure (B); (the smears of pancreatic specimen, original magnification ×200): individual nuclei were enlarged, chromatin was hyperchromatic, and the karyoplasm ratio was increased (C and D).

**Figure 2:**
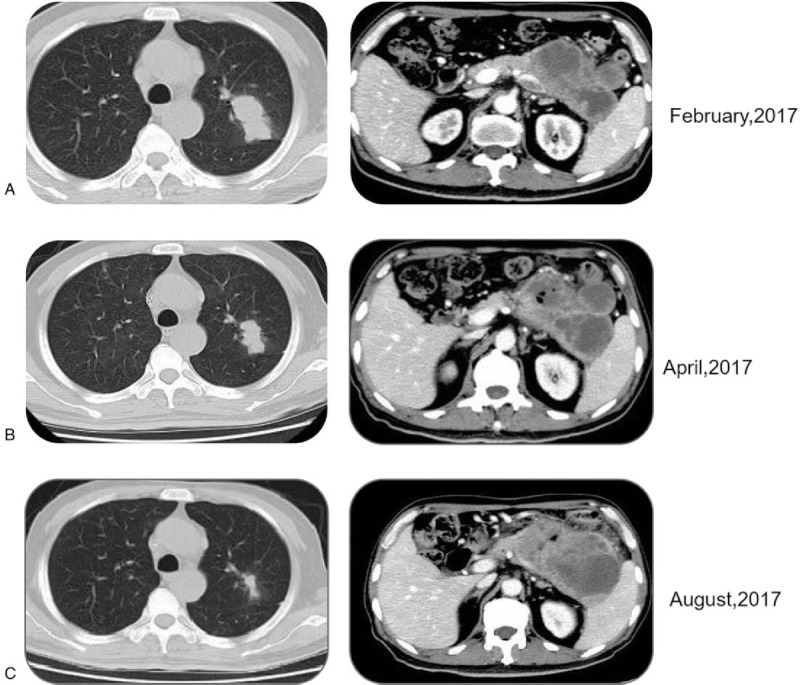
CT scans at baseline of AG treatment (A), after 2 cycles of AG (B), and after 6 cycles of AG (C).

**Figure 3:**
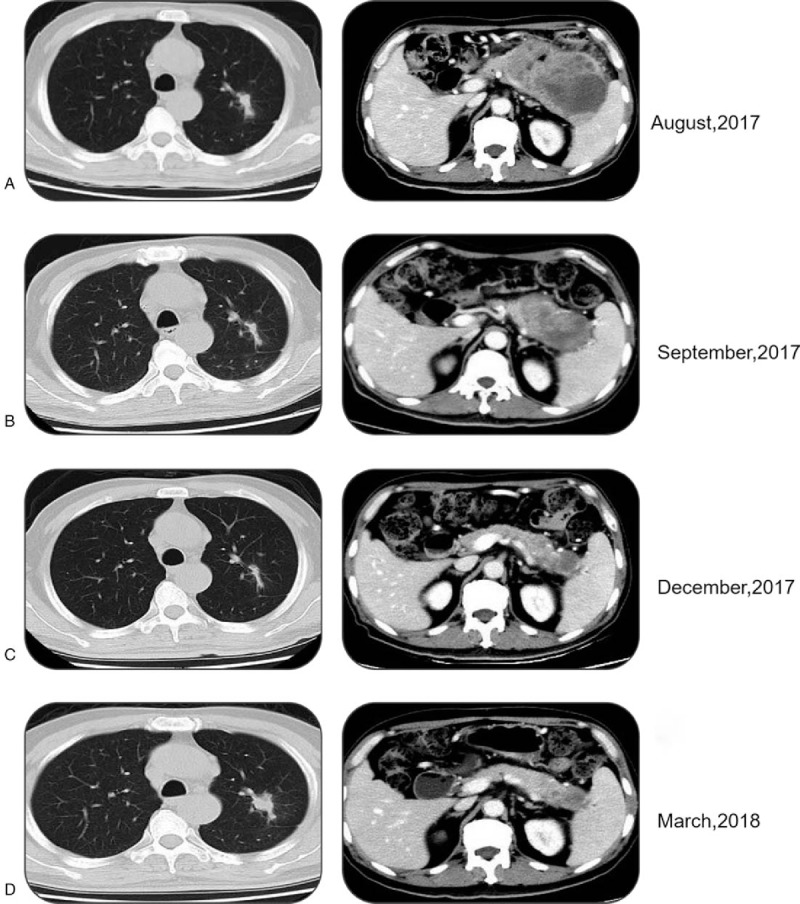
CT scans at baseline of Olaparib treatment (A), after 2 months of Olaparib treatment (B), after 4 months of Olaparib treatment (C), and after 7 months of Olaparib treatment (D).

**Figure 4:**
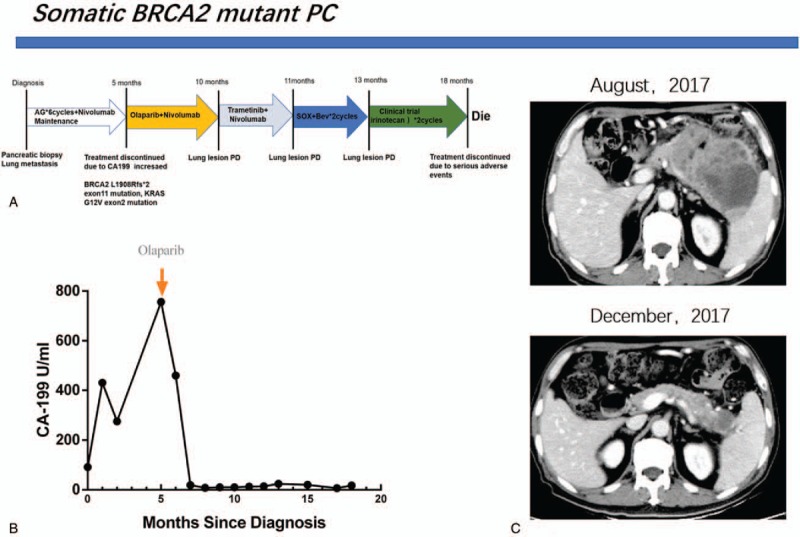
The process of treatment (A), the curve of CA199 and months since diagnosis (B), the CT scan at the baseline (August, 2017) and 4 months after the treatment of Olaparib (December, 2017), which was the optimal efficacy (C).

A 66-year-old male was suspected of a mass in the pancreas after a physical examination on June 9, 2015. A positron emission tomography-computed tomography (PET-CT) scan showed a large mass in the tail of the pancreas, 48 × 23 mm in size, which revealed an uneven density lower than that of the pancreatic parenchyma. The patient did not receive any form of therapy until December 2016. On December 5, 2016, a PET-CT reexamination showed a progressive enlargement of the large mass, which was 118 × 73.4 mm in size and was invading the retroperitoneum, gastric wall, splenic hilum, and partial spleen. Consequently, the patient received traditional Chinese medicine therapy. The patient was hospitalized after 1 month of persistent epigastric dull pain. A computed tomography (CT) scan indicated a large mass in the body and tail of the pancreas, and there were also signs of brain and lung metastasis. Results of an endoscopic ultrasonography-guided biopsy of the pancreatic mass revealed an adenocarcinoma, positive for CK7, CA-199, CK19, and Ki-67 (60%), but negative for CGA, SYN, and TTF1 according to immunohistochemistry. Then a needle biopsy from the left lung revealed an adenocarcinoma, which was infiltrating and possibly metastasizing on March 2, 2017. It was positive for CA199, but was negative for Napsin A, ALK, and TTF1 (Fig. 1). The patient was diagnosed with PC and classified as stage IV (T3NxM1) with histopathological and immunohistochemical examinations according to the 8th edition of the American Joint Committee on Cancer (AJCC) TNM staging for PC. The patient received palliative brain radiotherapy (300 cGy∗13F). After 1 month, the brain lesion was evaluated partial disease (PR). Meanwhile, serum CA-199 was elevated to a high level of 149.9 U/mL. Subsequently, he began receiving gemcitabine and albumin-bound paclitaxel chemotherapy from March 15, 2017 to June 30, 2017. After 6 cycles, the primary lesion of the pancreas was evaluated as a stable disease (SD), but PR in the lung lesion. At the patient's request, he received Nivolumab immunotherapy from May 24, 2017 as the maintenance therapy (180 mg every 2 week). The lesion was maintained at a stable state until August 2, 2017 using CT evaluation; however, serum CA-199 level was increased to 756.4 U/mL on August 1, 2017 (Fig. 2). With written informed consent, the specimen from the lung metastases, as well as a blood sample, was tested for a next-generation sequencing (NGS) panel for further treatment. The final report of NGS showed germline BRCA2 L1908Rfs∗2 exon11 mutation, KRAS G12 V exon2 mutation, TP53 R196∗ exon6 mutation, amplification of both the CCNE1 and GNA13 gene and tumor mutation burden 4.58 Muts/Mb. Therefore, Olaparib was orally administered from August 17, 2017 to March, 2018 at a dose of 300 mg twice a day. Serum CA-199 level descended to 460.0 U/mL after 1 month and remained at a normal level until September 7, 2018. The optimal efficacy of Olaparib treatment was PR until December 2017 (the lesion of the pancreas was classified as PR, and SD in the lung lesion). And there was no adverse effects. However, CT reexamination showed progressive enlargement of the lung, and new bone metastases were identified in March 2018. In summary, the overall efficacy of Nivolumab and Olaparib treatment was evaluated for progressive disease (PD), and its progression-free survival (PFS) was 7.4 months (Fig. 3). After genetic testing for a mutation in KRAS, he received Trametinib and Nivolumab immunotherapy from March 2018 (Trametinib at a dose 2 mg once a day, Nivolumab maintain original dose) to April 2018. A repeat CT revealed progressive enlargement of the lung on April 23, 2018. The overall efficacy was PD. Then he received SOX (Oxaliplatin and S-1) plus bevacizumab chemotherapy from April 26, 2018. Three cycles later the overall efficacy was evaluated for PD. Then the patient was enrolled in a trial with a clinical trial with irinotecan liposome injection for 2 cycles from June 28, 2018 to July 17, 2018. Because of the serious adverse events, the patient was removed from the clinical trial. Ultimately, the patient died from brain metastases on October 12, 2018. The overall survival (OS) was 18 months from the start of anticancer systemic therapy (Fig. 4).

## 3 Discussion

In this report, we present a rare case of unresectable advanced PC with lung, brain, and bone metastases, which was treated with standard systemic therapy. After the first line of therapy, the objective response rate (ORR) had a stable response (the primary lesion of the pancreas was evaluated as a SD, but PR in the lung lesion). By using Olaparib, the lesion of the pancreas was classified as PR, and SD in the lung lesion. The optimal efficacy of Olaparib treatment was PR and its PFS was 7.4 months, which indicated significant shrinkage.

PARP inhibitors provide a promising avenue of treatment for cancers associated with BRCA1/2 mutations.^[8]^ The phase III randomized POLO trial (NCT02184195), in which the effectiveness of maintenance Olaparib monotherapy following cisplatin, carboplatin, or oxaliplatin is being assessed, is currently in process. Study 42 is a clinical trial, which is used to assess the efficacy of oral Olaparib in patients with advanced cancer and who have a confirmed genetic BRCA1 and/or BRCA2 mutation. The tumor response rate for all 23 pancreatic patients was 21.7% (95% CI, 7.5–43.7), and stable disease persisting 8 weeks was observed in 34.8%(95% CI, 16.4–57.3). Overall median duration of response was 134 days, with 4.6 months in median PFS and 9.8 months in median OS.^[9]^ PARP inhibitors have previously been successfully combined with chemotherapy in other clinical settings. The clinical trial (NCT01296763) was a phase I clinical trial in unresectable PC patients. Moreover, the ORR for 18 patients was 23%, with 62% in the disease control rate (CR+PR+SD). One patient who was detected a BRCA2-mutation had a durable clinical response for >4 years.^[10]^ Cytotoxic T lymphocyte antigen 4 (CTLA-4), the programmed cell death protein-1/programmed death-ligand 1, and other immune checkpoint regulators have emerged as promising new targets for cancer therapeutics. MEDIOLOA and TOPACIO studies are trials that assess the efficacy and safety of Olaparib in combination with the immune system checkpoint inhibitors. The patients of MEDIOLOA trial received Olaparib for a 4-week run-in, followed by a combination of Olaparib and Durvalumab. The observed disease control rate (DCR) at 12 weeks was 81% through analysis of the results of the first 32 patients.^[11]^ TOPACIO is a phase I/II study that evaluates the safety and efficacy of combining treatments with the PARP inhibitor, niraparib, and pembrolizumab in patients who are diagnosed as triple-negative breast cancer or recurrent ovarian cancer. The ORR/DCR was 25%/68% in the 60 evaluable patients; and among the 11 tumor BRCA mutation evaluable patients, the ORR/DCR was 45%/73%.^[12,13]^ In addition, the efficacy of a combined regimen such as immunotherapy and chemotherapy for PC still requires further validation in the future. The targeted therapy Olaparib showed early signs of potential in treating PC in patients with mutations of the BRCA genes. With emerging therapeutic modalities and NGS development, it is increasingly relevant to consider mutation screenings of patients with PC.

In our study, the patient was treated with nivolumab and Olaparib, and a transient disease regression was observed. Is the outcome of the disease regression associated with immunotherapy? Could immunotherapy combining targeted therapy prolong the survival time? Currently, there is no evidence that supports the benefit of the application of immunotherapy combined with targeted drugs in PC. In recent years, immunotherapy has become increasingly popular in treating cancers, but the efficacy of such therapies is still being validated. It is difficult to conclude whether such combined therapies could help prolong the OS for such patients. However, in the future, additional clinical studies are still needed.

## Acknowledgments

The authors thank the Zhejiang Provincial Science and Technology Project (No. 2014C03040-2) and National Health and Family Planning Commission Research Fund & Zhejiang Provincial Medical and Health Major Science and Technology Plan Project (No. KWJ-ZJ-1802) for the support.

## Author contributions

**Conceptualization:** Huan Wang, Chenyu Mao, Nong Xu.

**Formal analysis:** Huan Wang, Nong Xu.

**Funding acquisition:** Nong Xu.

**Writing – original draft:** Huan Wang, Chenyu Mao, Liping Sun, Nong Xu.

**Writing – review & editing:** Huan Wang, Ning Li, Yulong Zheng, Nong Xu.
